# The diagnostic work up of growth failure in secondary health care; An evaluation of consensus guidelines

**DOI:** 10.1186/1471-2431-8-21

**Published:** 2008-05-13

**Authors:** Floor K Grote, Wilma Oostdijk, Sabine MPF De Muinck Keizer-Schrama, Paula van Dommelen, Stef van Buuren, Friedo W Dekker, Arnoldus G Ketel, Henriette A Moll, Jan M Wit

**Affiliations:** 1 Dept. of Paediatrics, Leiden University Medical Center, Leiden, The Netherlands; 2 Dept. of Paediatrics, Erasmus MC – Sophia Children's Hospital, Rotterdam, The Netherlands; 3 Dept. of Statistics, TNO Quality of life, Leiden, The Netherlands; 4 Dept. of Methodology & Statistics, University of Utrecht, The Netherlands; 5 Dept of Clinical Epidemiology, Leiden University Medical Center, Leiden, The Netherlands; 6 Dept. of Paediatrics, Spaarne Hospital, Haarlem, The Netherlands

## Abstract

**Background:**

As abnormal growth might be the first manifestation of undetected diseases, it is important to have accurate referral criteria and a proper diagnostic work-up. In the present paper we evaluate the diagnostic work-up in secondary health care according to existing consensus guidelines and study the frequency of underlying medical disorders.

**Methods:**

Data on growth and additional diagnostic procedures were collected from medical records of new patients referred for short stature to the outpatient clinics of the general paediatric departments of two hospitals (Erasmus MC – Sophia Children's Hospital, Rotterdam and Spaarne Hospital, Haarlem) between January 1998 and December 2002. As the Dutch Consensus Guideline (DCG) is the only guideline addressing referral criteria as well as diagnostic work-up, the analyses were based on its seven auxological referral criteria to determine the characteristics of children who are incorrectly referred and the adequacy of workup of those who are referred.

**Results:**

Twenty four percent of children older than 3 years were inappropriately referred (NCR). Of the correctly referred children 74–88% were short corrected for parental height, 40–61% had a height SDS <-2.5 and 21% showed height deflection (Δ HSDS < -0.25/yr or Δ HSDS < -1). In none of the children a complete detailed routine diagnostic work up was performed and in more than 30% no routine laboratory examination was done at all. Pathologic causes of short stature were found in 27 children (5%).

**Conclusion:**

Existing guidelines for workup of children with suspected growth failure are poorly implemented. Although poorly implemented the DCG detects at least 5% pathologic causes of growth failure in children referred for short stature. New guidelines for referral are required with a better sensitivity and specificity, wherein distance to target height should get more attention. The general diagnostic work up for short stature should include testing for celiac disease in all children and for Turner syndrome in girls.

## Background

Short stature or a poor growth rate can be the first manifestation of undetected diseases in children. Poor growth can be caused by a great diversity of congenital or acquired conditions, such as Turner syndrome, growth hormone deficiency (GHD) or celiac disease, for all of which early diagnosis and treatment are important. When treated at an early stage the effect on adult height is optimal and the quality of life will presumably improve. The possibility for proper treatment depends both on the early identification of these children in the community and on the accurate diagnostic work-up in the hospital afterwards.

For an early identification of children with abnormal growth it is important to have accurate and well-defined referral criteria, in combination with a good growth monitoring system. In a previous study we performed an inquiry about advised referral criteria among pediatric endocrinologists in Europe as well as in most industrialized countries around the world [1]. We concluded that there was little consensus. Moreover the literature provides only few guidelines for the analysis of short stature and these are based on consensus meetings rather than on experimental evidence [2,3]. In experimental studies on growth monitoring various arbitrary referral criteria were used [4-6].

There is not only scarce evidence on referral criteria, but also on the diagnostic work-up in secondary health care for children with poor growth. Although there are a number of consensus guidelines on the diagnosis of GHD [7-11] and some articles on the analysis of short stature in general [12-19], the articles are primarily expert-based reviews on how to deal with short stature rather than experimental studies on the outcome of laboratory investigations. Only one study evaluated the outcome of the analysis of short stature in a growth clinic, but in this study no standard protocol for the diagnostic work-up was used [20]. The only guideline reported so far that addresses the diagnostic work-up for short stature is the Dutch Consensus Guideline (DCG) [3].

The DCG was prepared in 1996, containing a section on referral criteria and a section on diagnostic procedures. Its implementation consisted of a single publication in a Dutch medical journal, a book and a couple of courses [3,21]. It is not known how many doctors are aware of the guideline and whether or not it changed medical practices.

In the present paper we wished to assess how many children were correctly referred to secondary health care according to the DCG, to evaluate the diagnostic work-up in secondary health care and to study the frequency of underlying medical disorders.

## Methods

We performed a retrospective observational study in the outpatient clinics of the general paediatric departments of both a university hospital (Erasmus MC – Sophia Children's Hospital, Rotterdam) and a general hospital (Spaarne Hospital, Haarlem). In both clinics the DCG was well known and used during the study period. All new patients referred for short stature between January 1998 and December 2002 were identified retrospectively. The children in whom the cause of growth retardation was already known were excluded. A previously described problem-orientated patient classification system [22] was used to identify the children in the university hospital. In the general hospital the children were identified by a local registration system, consisting of a hand written registry of reasons for referral of all new patients.

The following information was obtained from the medical records: date of birth, date of first presentation at the outpatient clinic, gender, ethnicity, perinatal information (birth weight, length, gestation, maternal obstetric problems etc.), family history, clinical presentation (symptoms and signs), information on puberty, longitudinal height measurements until the first presentation at the outpatient clinic, laboratory test results, radiological and pathological evaluations and final diagnosis. If the ethnicity was not recorded, it was assessed based on the patient's first and family name according to an algorithm reported earlier [23].

The DCG addresses five stages in the analysis of short stature. First of all it focuses on seven auxological referral criteria (Table 1). When a child is referred according to these criteria, the paediatrician is subsequently advised to follow four diagnostic steps:

• The patient's history, the physical examination, growth data and a hand radiograph should be collected to determine signs or symptoms that may indicate a specific disease.

• In the presence of specific clinical clues, appropriate further specific investigations are done. When there are no signs or symptoms leading to the suspicion of a certain disease, a list of laboratory investigations is advised for screening of several pathological conditions (Table 2).

• Dependent on the abnormalities in the screening laboratory investigations further, more specific tests can be performed to establish the final diagnosis.

• If there is no indication of a certain disease after the preceding procedures the three following tests should still be considered: chromosomal analysis for Turner syndrome in girls, a biopsy to prove or rule out celiac disease and the determination of zinc to investigate zinc deficiency in children with failure to thrive [24].

**Table 1:** Seven auxological referral criteria taken from the Dutch Consensus Guideline [3].

***Description rule***		***Criteria***	***Rule nr*.**
Absolute height		HSDS* < -2.5	1
Clinical symptoms		HSDS* < -1.3 AND (dysmorphic features OR disproportions)	2
Persistent short stature after born SGA**		SGA** AND HSDS* < -1.88 after the age of 2 years	3
Short for target height and population (HSDScorr)	♂: < 10 yr and > 13.4 yr;	HSDS* < -1.3 AND HSDS-THSDS^§ ^< -1.3	4
	♀: < 9 yr and > 12.3 yr		
	Pubertal age^‡^: ♂: 10 – 13.4 yr; ♀: 9 – 12.3 yr	HSDS* < -1.3 AND HSDS-THSDS^§ ^< -1.3 AND pubertal signs (♂: genit ≥ Tanner stage 2 OR testis volume ≥ 4 ml; ♀: breast ≥ Tanner stage 2)	5
Height deflection^†^	♂: < 10 yr and > 13.4 yr; ♀: < 9 yr and > 12.3 yr	T2 – T1 > 1 (SDS1 – SDS2)/(T2-T1) < -0.25 OR (SDS1 – SDS2) < -1	6
	Pubertal age^‡^: ♂: 10 – 13.4 yr; ♀: 9 – 12.3 yr	T2 – T1 > 1 (SDS1 – SDS2)/(T2-T1) < -0.25 OR (SDS1 – SDS2) < -1 With pubertal signs	7

* HSDS = Height Standard Deviation Score (Height – mean height for the same age and sex/SD for the same age and sex)

**SGA = Small for gestational age

^§ ^THSDS= Target Height Standard Deviation Score (Target height = (height of mother (+height of father +13) + 4.5)/2)

^† ^Height deflection: Height deflection is formulated as Delta HSDS < -0.25 per year OR a delta HSDS <-1SDS over a longer period (not specified).

^‡ ^Pubertal age: When a child does not show any pubertal signs (♂: genit ≥ Tanner stage 2 OR testis volume ≥ 4 ml; ♀: breast >= Tanner stage 2) at this age referral is not necessary.

**Table 2:** Laboratory investigations in the diagnostic work up according the DCG

***Laboratory investigations***	***In order to diagnose***	***Category***
***Blood***		
Hb, Ht, Leukocytes, Cell indices, Leukocyte differentiation, ESR (Ferritin)	Anemia/infections (and celiac disease and cystic fibrosis)	I
ALAT, ASAT, γ GT	Liver diseases	II
Albumin, Creatinine, Sodium, Potassium, Calcium, Phosphate, Alkaline phosphatase, acid-base equilibrium	Renal diseases	III
IgA-anti endomysium, IgA- antigliadin, Anti-tissue glutaminase*, Total IgA	Celiac disease	IV
TSH, FT4	Hypothyroidism	V
IGF-I	Growth hormone deficiency	VI
FSH**	Turner syndrome	VII
***Urine***		
pH, glucose, protein, blood and sedimentation	Renal diseases	VIII

*At the moment the consensus meeting took place, anti tissue glutaminase as a diagnostic tool for celiac disease was not yet introduced nation wide.

**Only in girls.

For the evaluation of the diagnostic work up in this project all stages were taken into account. The auxological criteria as mentioned in the DCG were used to determine whether the children were correctly referred to the outpatient clinic. Since children under the age of three years may not yet show a stable growth pattern but are still seeking their individual growth channel (expressed as percentile or standard deviation score (SDS) position), most rules (1, 3–7) were not strictly applicable to this age group. Therefore it was decided to analyse this group separately. For the analyses we allowed a child to meet several criteria at the same time. Although plasma FSH in girls is only of diagnostic value in girls younger than 2 and older than 9 years, we analysed all ages in girls for this test, since there is no such specific recommendation in the current consensus.

All data were analysed in SPSS version 11. Height SDS was calculated using the 1997 Dutch reference growth data [25]. Small for gestational age (SGA) was defined as a birth weight and/or length SDS < -2 for gestational age, compared to recent Swedish reference values [26]. Differences between the two hospitals were calculated using the chi-square test.

The study was approved by the Medical Ethical Committees of the Leiden University Medical Center, the Erasmus MC – Sophia Children's Hospital, Rotterdam and the Spaarne Hospital, Haarlem.

## Results

### Patients

**Figure 1:**
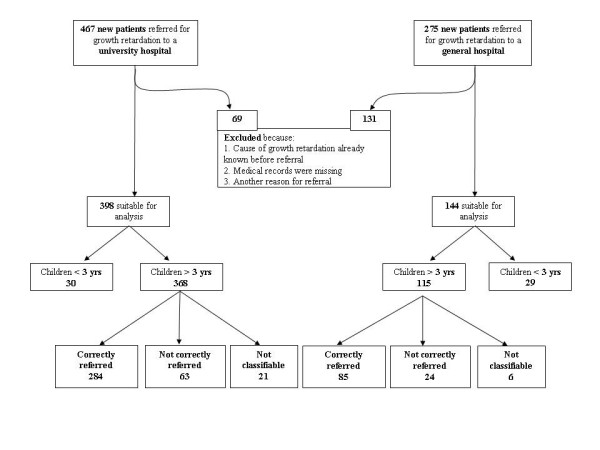
Population description.

Between January 1998 and December 2002, 742 children were referred to the two hospitals for short stature (university hospital: n = 467, general hospital: n = 275) (see Fig. 1). Two hundred children were excluded either because the cause of growth retardation was already known before referral, the medical records were missing or there was another reason for referral than short stature. Hence, 542 cases were suitable for analysis. Fifty-nine children were under the age of 3 years at time of referral and were analysed separately. According to referral criteria mentioned in the DCG 76.4% (77.1% in the university and 73.9% in the general hospital) of the children older than 3 years were correctly referred (CR). In 5.6% children (5.7% in the university and 5.2% in the general hospital) there was insufficient information to assess whether the referral met the criteria (not classifiable (NC)) and in 18.0% the children did not meet the referral criteria (not correctly referred, NCR) according to the DCG (17.1% in the university and 20.9% in the general hospital).

Details of all patients are shown in Table 3. The study groups were significantly different between the two hospitals for gender, ethnicity, height SDS (HSDS) at time of referral and target height.

**Table 3:** General characteristics of the study population in the university hospital (n = 398) and the general hospital (n = 144)

	**University hospital**	**General hospital**	**Difference between 2 hospitals **(p-value)
Gender Male: n (%)	219 (55%)	65 (45%)	0.04
Ethnicity N (%)			
Dutch/European	291 (73.1%)	127 (88.2%)	0.01
Turkish	31 (7.8%)	6 (4.2%)	
Moroccan	11 (2.8%)	1 (0.7%)	
Others:	54 (13.6%)	10 (6.9%)	
Unknown	11 (2.8%)	0 (0%)	
Age at time of referral (yrs): mean (SD)	9.1 (4.0)	8.4 (4.7)	0.2
HSDS at time of referral: mean (SD)	-2.3 (0.8)	-1.9 (0.9)	< 0.01
THSDS: mean (SD)	-0.7 (1.0)	-0.2 (0.9)	< 0.01
HSDS – THSDS at time of referral: mean (SD)	-1.6 (0.8)	-1.7 (1.0)	0.7
Delta HSDS in last year before referral: mean (SD)	-0.08 (0.3)	-0.10 (0.3)	0.5
Dysmorphic features: n (%)	22 (5.5%)	2 (1.4%)	0.1
Disproportion: n (%)	15 (3.8%)	2 (1.4%)	0.08

HSDS = height standard deviation score; THSDS = target height standard deviation score

### Referral criteria

Table 4 shows how many children in the correctly referred group complied with the 7 different referral criteria mentioned in the DCG (although longitudinal data were used for the analyses a child could only meet a specific referral criterion once). There was a significant difference in referral pattern between the two hospitals with respect to absolute height and "short for target height and population" (HSDScorr). In both hospitals HSDScorr is the criterion most complied with, followed by absolute height and height deflection.

**Table 4:** Auxological criteria applicable to the correctly referred (CR) group of children > 3 years old in both the university hospital (n = 284) and the general hospital (n = 85) (multiple criteria per patient are possible).

**Description rule**		**Rule nr**.	**University hospital**	**General hospital**	**Difference between 2 hospitals **(p-value)
				
			**n (%)**	**n (%)**	
Absolute height		*1*.	173 (60.9)	34 (40.0)	0.01
Clinical symptoms		*2*.	23 (8.1)	3 (3.5)	0.1
Persistent short stature after born SGA		*3*.	55 (19.4)	13 (15.3)	0.3
Short for target height and population (HSDScorr)	♂: < 10 yr and > 13.4 yr; ♀: < 9 yr and > 12.3 yr	*4*.	210 (73.9)	75 (88.2)	0.04
	♂: 10 – 13.4 yr; ♀: 9 – 12.3 yr	*5*.	9 (3.2)	0 (0)	
Height deflection	♂: < 10 yr and > 13.4 yr; ♀: < 9 yr and > 12.3 yr	*6*.	58 (20.4)	18 (21.1)	0.9
	♂: 10 – 13.4 yr; ♀: 9 – 12.3 yr	*7*.	0 (0)	0 (0)	

### Diagnostic work-up after referral (Table 5)

**Table 5:** Diagnostic work up in correctly (CR) and not correctly referred (NCR) children > 3 years old.

		**University hospital**		*General hospital*		*Difference in categories between 2 hospitals **(p-value)***
			
		**CR (n = 284) (%)**	**NCR (n = 63) (%)**	**CR (n = 85) (%)**	**NCR (n = 24) (%)**	
**Routine laboratory investigations**						
Anemia/infections		0.4/*39.8*	0.0/*25.4*	1.2/57.6	4.2/*37.5*	< 0.01
Liver diseases		22.5/*4.2*	2.8/*0.0*	14.1/*21.2*	8.3/*25.0*	0.03
Renal diseases (blood)		1.4/*27.3*	0.0/*17.5*	0.0/*38.8*	0.0/*33.3*	0.5
Celiac disease		21.1/*15.8*	11.1/*3.2*	49.4/*4.7*	25.0/*4.2*	< 0.01
Hypothyroidism		37.0/*3.2*	2.5/*0.0*	45.9/*3.5*	29.2/*8.3*	0.02
Growth hormone deficiency		35.2	9.5	45.9	25	0.02
Turner syndrome *		17.2	6.5	14.0	18.1	1.0
Renal diseases (urine)		0.0/*19.4*	0.0/*19.0*	0.0/*40.0*	0.0/*29.2*	< 0.01
Combined categories (at least one test category)		4.9/*52.1*	1.6/*39.7*	18.8/*49.4*	33.3/*16.7*	< 0.01
**Further and specific diagnostics**						
Special investigations	Chromosomal analyses for Turner syndrome*	26.2	6.5	26.0	0.0	0.9
	Biopsy**	2.1	0.0	5.9	0.0	0.03
	Zinc-determination	0.4	0.0	0.0	0.0	0.6
Further, more specific tests	GH-tests	16.2	3.2	12.9	0.0	0.3
	Other tests***	8.8	3.2	28.2	25.0	< 0.01

Percentages of patients with Complete (all elements)/partial (at least one element) evaluation are given (several test categories per patient).

CR = Correctly referred; NCR = Not correctly referred

* These categories are only applicable to girls. The percentages are therefore calculated only on the female population.

**biopsy to rule out celiac disease.

*** other tests, like stool examinations, X-rays of the skeleton, ultrasounds of abdomen, hart, kidneys and thyroids, serum levels of steroids, genetic analyses, immunologic tests and allergic tests

In 43% of the correctly referred patients in the university hospital and 32% in the general hospital, no routine laboratory examination was done at all. In 52% and 49%, respectively, only some of the categories mentioned in the laboratory screening of the DCG were covered and in only 5% and 19% respectively, all categories were covered. In none of the children a complete detailed routine diagnostic work up was performed. There was a significantly different approach towards the diagnostic workup between the two hospitals. In the general hospital more tests were performed, with less distinction between the correctly referred group and the non-correctly referred group. This difference was significant for all categories in the routine laboratory investigations except for the investigations for renal diseases in blood and screening for Turner syndrome. Significantly more biopsies to rule out celiac disease and other, more specific tests were done in the regional hospital. Less than a quarter of the girls were screened on FSH for Turner syndrome in the correctly referred group and in approximately 26% chromosomal analysis for Turner syndrome was performed. When the age rules recommended by paediatric endocrinologists (plasma FSH only < 2 years and > 9 years, see material and methods) were applied the figures hardly changed. The determination of zinc was used only once in the diagnostic work up.

### Outcome

In 80 children (14.8%) the diagnosis of persistent short stature after born SGA could be made on the basis of recorded birth size, although only 17 children (3.3%) were classified as such by the physicians. Pathologic causes of short stature were found in 27 children (5%) (see Table 6). A large share of these were due to Turner, GHD, and celiac disease. Other pathological causes were: syndromes (n = 2: Noonan syndrome, Leri Weill syndrome), anaemia (n = 3), skeletal diseases (n = 4) and emotional deprivation (n = 1). Three children born SGA had also a pathologic cause for short stature (celiac disease, Turner syndrome and GHD). These three were classified under their pathologic cause and not under SGA in Table 6.

**Table 6:** Diagnoses after diagnostic workup of short stature.

	**University hospital**				**General hospital**				**Total**
			
	**Children < 3 years**	**Children > 3 years**			**Children < 3 years**	**Children > 3 years**			
					
		**CR**	**NCR**	**NC**		**CR**	**NCR**	**NC**	
					
	**n = 30**	**n = 284**	**n = 63**	**n = 21**	**n = 29**	**n = 85**	**n = 24**	**n = 6**	
	**n (%)**	**n (%)**	**n (%)**	**n (%)**	**n (%)**	**n (%)**	**n (%)**	**n (%)**	**n = 542 (%)**

GHD	1	6	0	0	0	0	0	0	7
CD	0	1	0	0	3	2	0	1	7
Turner	0	2	0	0	0	1	0	0	3
Other pathology	4	5	1	0	0	0	0	0	10
Total pathology	5 (16.7)	14 (5.2)	1 (5.7)	0 (0)	3 (10.3)	3 (3.4)	0 (0.0)	1 (16.7)	27 (5.0)
SGA only	4 (13.3)	54 (20.1)	3 (3.5)	4 (18.2)	1 (3.4)	13 (14.9)	0 (0.0)	1 (16.7)	80 (14.8)
Idiopathic	21 (70)	216 (74.7)	59 (90.8)	17 (81.8)	25 (86.2)	69 (81.6)	24 (92.1)	4 (66.8)	435 (80.2)

CR = Correctly referred; NCR = Not correctly referred; NC = Not classifiable (information to confirm CR was lacking);

SGA = Small for gestational age (with persistent short stature after two years); GHD = Growth hormone deficiency; CD = Celiac disease

Of all 27 children with pathologic outcome, seven were referred for other reasons (anaemia (2), coughing (2), delayed closure of fontanel (1), health check after adoption (1), poor weight gain (1) and poor food intake (1)) in addition to their short stature. Five children had dysmorphic features at the time of referral (2 children with Turner syndrome, 1 child with Noonan, 1 child with achondroplasia and 1 child with partial GHD) and 3 children were disproportionate (2 children with achondroplasia and 1 child with Leri- Weill syndrome). Six children had already been seen by a specialist before referral: 2 for short stature (they were referred for a second opinion), 1 for hydrocephalus, 1 for exostoses, 1 for neurofibromatosis and glioma of the medulla oblongata and 1 for ASD, but none of the children were previously investigated for short stature. For none of the children the family or medical history was helpful in determining the cause. Most of the correctly referred children with pathology complied with a deviant HSDScorr (83.4%), followed by absolute height (see Table 7). The only child with a pathologic cause that was incorrectly referred, had a height SDS of -1.7 SDS at time of referral and was referred because of its short stature in combination with an undefined anaemia. The child turned out to have a beta-thalassemia.

**Table 7:** Auxological criteria applicable to children with pathology < 3 yrs (n = 8, excluding SGA only) and correctly referred (CR) children with pathology > 3 yrs (n = 17, excluding SGA only) (multiple criteria per patient are possible).

**Description rule**		**Rule nr**.	**< 3 yrs n (%)**	**> 3 yrs n (%)**
Absolute height		*1*.	7 (87.5)	11 (64.7)
Clinical symptoms		*2*.	2 (25.0)	5 (29.4)
Persistent short stature after born SGA		*3*.	0 (0.0)	3 (17.6)
Short for target height and population (HSDScorr)	♂: < 10 yr and > 13.4 yr; ♀: < 9 yr and > 12.3 yr	*4*.	6 (75.0)	15 (88.2)
	♂: 10 – 13.4 yr; ♀: 9 – 12.3 yr	*5*.		
Height deflection	♂: < 10 yr and > 13.4 yr; ♀: < 9 yr and > 12.3 yr	*6*.	2 (25.0)	3 (17.6)
	♂: 10 – 13.4 yr; ♀: 9 – 12.3 yr	*7*.		

## Discussion

This study was designed to evaluate consensus guidelines on poor growth in secondary health care and their outcome in terms of pathology. According to the referral criteria mentioned in the DCG 76.4% (284 in the university and 85 in the general hospital) of the children older than 3 years were correctly referred. In both hospitals "short for target height and population (HSDScorr)" appears to be the criterion most complied with, followed by absolute height and height deflection. The approach towards the diagnostic workup was significantly different between the two hospitals, but in none of the children a complete detailed routine diagnostic work up was performed. Pathologic causes for short stature were found in 27 children (5%) and in 80 children (14.8%) the short stature was classified as persistent short stature after born SGA.

The Netherlands has a health care system based on referral, in which outpatient clinics of general paediatric departments mainly provide secondary health care to referred patients from the well organised national primary health care system. Consequently we believe that by identifying all children referred for short stature to outpatient clinics most children with short stature from the two geographical areas were gathered. The majority of the children older than 3 years were referred correctly according to the DCG. However, if the DCG had been followed strictly, more than 38% of the normal population of children would have been referred, as van Buuren et al pointed out in their study [27].

The 5% of pathology found in our study concurs with previous reports [4,6,28]. In the Wessex growth study 8 children (4.4%) were identified as having an organic disease among the 180 children, whose height on screening at school entry was below the 3^rd ^percentile [6]. In the Oxford study Ahmed et al reported 7 newly recognized children (3.0%) with organic disease among the 260 children whose height was below – 2 SDS, measured at the ages of 3 and 4.5 years [4]. In the Utah growth study [29] twenty-five out of 555 children (4.5%) were newly discovered as having GHD, hypothyroidism or Turner syndrome and another 53 children (9.5%) had other medical reasons for their poor growth (height below the 3^rd ^percentile and/or growth rate below 5 cm/yr). In contrast to these population based studies Grimberg et al and Green et al found a higher percentage of newly diagnosed children with organic causes for their poor growth (23.7% (66 out of 278 children and 40% (79 out of 198), respectively) [20,29]. The children included in these studies were however referred to specialised growth centres because of short stature, without specific choices of anthropometric indicators or criteria for abnormality.

In both hospitals most children with pathology older than three years complied with the HSDScorr rule, followed by the absolute height rule and height deflection rule. This result is contrary to most findings in literature, where absolute height is referred to as the most important criterion for abnormal growth [1,2]. It concurs however with the observation by Van Buuren et al that the best decision rule to detect children with Turner syndrome, one of the major causes of short stature, is the distance between height SDS and target height [30]. In the 8 children with pathology referred before the age of 3 years (CD (n = 3), GHD (n = 1), anaemia (n = 2), skeletal diseases (n = 2) and emotional deprivation (n = 1)) both HSDScorr and absolute height seemed important criteria. According to the English consensus these children would however not have been diagnosed at this point, as it recommends a single measurement at the age of 5 years old [2]. As these children did not reach the age of 5 years in our study we are not able to evaluate whether they would have been picked up in the English system, but we can surely say that there would have been a delay. Especially in the children with GHD and celiac disease early diagnosis and treatment is important for its prognosis. In order to improve the referral criteria for growth monitoring with optimal cut-off points, we believe that more studies, similar to the recent report on Turner syndrome are required, with specific attention for children under the age of 3 years.

As far as we know, the DCG is the only published guideline on the general diagnostic work-up for short stature in secondary health care. Despite the fact that this consensus was well known in both hospitals participating in this study, in none of the children a complete detailed routine diagnostic work up as proposed in the DCG was performed and in 43% of the patients in the university hospital and 32% in the general hospital no routine laboratory examination was done at all. The heterogeneity of tests used in the diagnostic work up, resulting in many missing data for the individual tests in this study, does unfortunately not allow us to construct an evidence-based decision rule for the general diagnostic work-up in children with short stature. We know however from a previous study that testing on celiac disease should be part of this work up, especially when there is no specific indication of another cause for short stature [31]. Likewise the diagnosis of Turner syndrome should be considered in any girl with unexplained short stature [32,33]. In contrast, the prior-probability of CF in infants or children with a low weight or length for age is very low and therefore a sweat test is not necessary in all children with short stature (Van Dommelen et al, submitted). Whether an acid-base equilibrium is necessary in every child with short stature to rule out renal acidosis will be addressed in a later study. In the meantime, the available evidence so far can be used to construct a new guideline with an expected acceptable efficacy and efficiency.

## Conclusion

For the identification of children with abnormal growth accurate and well-defined referral criteria and a diagnostic work up are important. The current study shows that with the DCG, though only partially adhered to, at least 5% pathologic growth failure could be detected. In a substantial part of these children (30%) there would at least have been a delay in diagnosis if the English consensus guideline would have been used. From previous studies it is known on the other hand that the DCG leads to too many referrals [27]. Therefore new guidelines are needed with a better sensitivity and specificity, in which target height should play a more prominent role. We have recently proposed such guidelines [34]. Furthermore, the implementation process should receive more attention Concerning the general diagnostic work up for short stature we emphasise the importance of testing for celiac disease in all children and for Turner syndrome in girls.

## Competing interests

The authors declare that they have no competing interests.

## Authors' contributions

All authors contributed to the planning, the design of the study and read and approved the final manuscript. FKG, AGK and HAM were responsible for the collection of the data. All the other authors participated together with FKG in the analytical part. FKG wrote the manuscript.

## Pre-publication history

The pre-publication history for this paper can be accessed here:



## References

[B1] F.K. G, Oostdijk W, de Muinck Keizer-Schrama SMPF, Dekker FW, Verkerk PH, Wit JM (2005). Growth monitoring and diagnostic work-up of short stature: an international inventorisation.. J Pediatr Endocrinol Metab.

[B2] Hall DM (2000). Growth monitoring. Arch Dis Child.

[B3] Muinck Keizer-Schrama SM (1998). [Consensus 'diagnosis of short stature in children.' National Organization for Quality Assurance in Hospitals]. Ned Tijdschr Geneeskd.

[B4] Ahmed ML, Allen AD, Sharma A, Macfarlane JA, Dunger DB (1993). Evaluation of a district growth screening programme: the Oxford Growth Study. Arch Dis Child.

[B5] Frindik JP, Kemp SF, Kearns FS, Hale B (1992). Growth screening. A positive medical experience. Clin Pediatr (Phila).

[B6] Voss LD, Mulligan J, Betts PR, Wilkin TJ (1992). Poor growth in school entrants as an index of organic disease: the Wessex growth study. BMJ.

[B7] (2000). Consensus guidelines for the diagnosis and treatment of growth hormone (GH) deficiency in childhood and adolescence: summary statement of the GH Research Society. GH Research Society. J Clin Endocrinol Metab.

[B8] Hilken J (2001). Uk audit of childhood growth hormone prescription, 1998. Arch Dis Child.

[B9] Juul A, Bernasconi S, Chatelain P, Hindmarsh P, Hochberg Z, Hokken-Koelega A, SM MKS, Kiess W, Oberfield S, Parks J, Strasburger CJ, Volta C, Westphal O, Skakkebaek NE (1999). Diagnosis of growth hormone (GH) deficiency and the use of GH in children with growth disorders. Horm Res.

[B10] Juul A, Bernasconi S, Clayton PE, Kiess W, DeMuinck-Keizer SS (2002). European audit of current practice in diagnosis and treatment of childhood growth hormone deficiency. Horm Res.

[B11] Wilson TA, Rose SR, Cohen P, Rogol AD, Backeljauw P, Brown R, Hardin DS, Kemp SF, Lawson M, Radovick S, Rosenthal SM, Silverman L, Speiser P (2003). Update of guidelines for the use of growth hormone in children: the Lawson Wilkins Pediatric Endocrinology Society Drug and Therapeutics Committee. J Pediatr.

[B12] Cappa M, Loche S (2003). Evaluation of growth disorders in the paediatric clinic. J Endocrinol Invest.

[B13] Duck SC (1996). Identification and assessment of the slowly growing child. Am Fam Physician.

[B14] Fox LA, Zeller WP (1995). Evaluation of short stature. Compr Ther.

[B15] Goldberg MJ, Yassir W, Sadeghi-Nejad A, Stanitski CL (2002). Clinical analysis of short stature. J Pediatr Orthop.

[B16] Halac I, Zimmerman D (2004). Evaluating short stature in children. Pediatr Ann.

[B17] Hermanussen M (1998). The analysis of short-term growth. Horm Res.

[B18] Hindmarsh PC, Brook CG (1988). Auxological and biochemical assessment of short stature. Acta Paediatr Scand Suppl.

[B19] Rosenfield RL (1996). Essentials of growth diagnosis. Endocrinol Metab Clin North Am.

[B20] Green AA, Macfarlane JA (1983). Method for the earlier recognition of abnormal stature. Arch Dis Child.

[B21] de Muinck Keizer-Schrama S.M.P.F. BFSOWRB (1998). Diagnostiek kleine lichaamslengte bij kinderen.

[B22] Van Steensel-moll HA, Jongkind CJ, Aarsen RS, De Goede Bolder A, Dekker A, van Suijlekom-Smit LW, Smit M, Kraayenoord S, Derksen-Lubsen G (1996). Een probleemgeorienteerd patientenclassificatiesysteem voor de algemene kindergeneeskunde II.. Tijdschr Kindergeneeskunde.

[B23] Bouwmeester-Landweer MBR (2006). Early home visitation in families at risk for maltreatment. Phd Thesis.

[B24] Kaji M, Nishi Y (2006). Growth and minerals: Zinc. Growth, Genetics & Hormones.

[B25] Fredriks AM, Buuren van S, Burgmeijer RJF, Meulmeester JF, Beuker RJ, Brugman E, Roede MJ, Verloove-Vanhorick SP, Wit JM (2000). Continuing positive secular growth change in the Netherlands 1955-1997. Pediatric Research.

[B26] Niklasson A, Ericson A, Fryer JG, Karlberg J, Lawrence C, Karlberg P (1991). An update of the Swedish reference standards for weight, length and head circumference at birth for given gestational age (1977-1981). Acta Paediatr Scand.

[B27] Buuren van S, Bonnemaijer-Kerckhoffs DJ, Grote FK, Wit JM, Verkerk PH (2004). Many referrals under Dutch short stature guidelines. Arch Dis Child.

[B28] Lindsay R, Feldkamp M, Harris D, Robertson J, Rallison M (1994). Utah Growth Study: growth standards and the prevalence of growth hormone deficiency. J Pediatr.

[B29] Grimberg A, Kutikov JK, Cucchiara AJ (2005). Sex differences in patients referred for evaluation of poor growth. J Pediatr.

[B30] Buuren van S, Dommelen van P, Zandwijken GR, Grote FK, Wit JM, Verkerk PH (2004). Towards evidence based referral criteria for growth monitoring. Arch Dis Child.

[B31] Rijn van JC, Grote FK, Oostdijk W, Wit JM (2004). Short stature and the probability of coeliac disease, in the absence of gastrointestinal symptoms. Arch Dis Child.

[B32] Davenport ML, Punyasavatsut N, Stewart PW, Gunther DF, Savendahl L, Sybert VP (2002). Growth failure in early life: an important manifestation of Turner syndrome. Horm Res.

[B33] Moreno-Garcia M, Fernandez-Martinez FJ, Barreiro ME (2005). Chromosomal anomalies in patients with short stature. Pediatr Int.

[B34] Grote FK, van Dommelen P, Oostdijk W, SM MKS, Verkerk PH, Wit JM, van Buuren S (2008). Developing evidence-based guidelines for referral for short stature. Arch Dis Child.

